# The Constitutive Relationship for Corroded Steel Bars: Model and Analysis

**DOI:** 10.3390/ma12244058

**Published:** 2019-12-05

**Authors:** Cheng Xiong, Chaoqun Zeng, Yanru Li, Ly Li, Ping Li, Dawang Li

**Affiliations:** Guangdong Provincial Key Laboratory of Durability for Marine Civil Engineering, Shenzhen University, Shenzhen 518060, Chinachaoqun.zeng@szu.edu.cn (C.Z.);

**Keywords:** steel corrosion, reinforcement, constitutive relation, analytical model, slotted steel specimen

## Abstract

Combining the theoretical derivation and numerical calculations, the characteristic changes of the tensile constitutive relation of corroded steel bars and their underlying mechanisms are studied. Corroded steel bars are regarded as a combination of three parts, which include uncorroded part, corroded part with variable cross section, and uniform corroded part. It is assumed that in all three parts the steel material follows a simplified trilinear constitutive relation of a mild steel material (elasticity, yielding, and hardening), from which an analytical model describing the overall tensile constitutive relation of the corroded steel bar is developed. Based on the experimental data of slotted steel bars, the validation of the present analytical model is provided. The results show that the trilinear model can give relatively accurate prediction of the characteristic parameters of corroded steel bars. The influences of corrosion rate on the mechanical properties of corroded steel bars are examined using the proposed model.

## 1. Introduction

Corrosion of steel bars in a reinforced concrete structure could cause deterioration of the overall performance of the structure [1,2,3]. Compared to uncorroded steel bar, the corroded steel bar has a reduced cross-sectional area and the sectional area reduction is normally not uniformly distributed along its longitudinal direction [4,5,6,7]. Simultaneously, its mechanical properties, such as the overall strength and ductility of a corroded steel bar, are heavily impacted by the increase of corrosion degree [8,9,10]. There are many studies on the constitutive characteristics of corroded steel bars, but most of them are based on experimental results to give empirical estimation on the effect of corrosion on the mechanical properties of corroded steel bars [11,12,13,14,15,16]. Owing to the complex and variable appearance of corroded steel bars [17,18,19,20], it is still not fully understood about how various parameters affect the constitutive relation of corroded steel bars. Therefore, establishing an analytical model of the constitutive relationship of a corroded steel bar is an effective way to explore the problem.

Establishing an analytical model requires some basic premises. Based on the assumption of cross-sectional area reduction caused by corrosion in corroded steel bars, Li et al. [21] derived a bilinear constitutive relationship for corroded steel bars. The analytical formula facilitates regularity analysis and has predictive accuracy for the stress and strain of the yield point and ultimate point of corroded steel bars.

This paper is the further development of the analytical model proposed in [21], using a more accurate constitutive relation of steel materials and considering more general corrosion profile occurring in steel bars. First, the bilinear material constitutive model is replaced with trilinear material model containing the yielding platform, which is more consistent with the constitutive curve of actual mild steel materials. Second, a corroded part with variable cross section is added between the uncorroded part and the uniform corroded part, which provides a more realistic corrosion pattern for corroded steel bars.

## 2. Assumptions and Model of Steel Bars

### 2.1. Basic Model of Steel Bars

**Figure 1:**
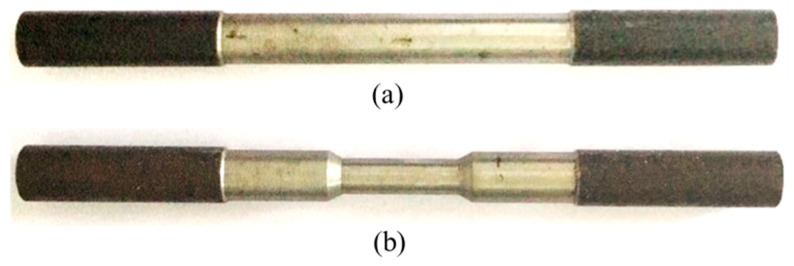
Specimens for uncorroded steel bars (**a**) and for corroded steel bars (**b**).

Figure 1 shows two idealized types of specimens: one represents the uncorroded reinforcing steel bars, and the other represents the corroded reinforcing steel bars for which the cross-section area reduction is assumed to be caused by corrosion. The former has uniform cross-section; whereas the latter has three different cross-section parts (no corroded part, uniformly corroded part, and linearly varied from no corroded part to uniformly corroded part). Note that the two end portions (dark color) are for the purpose of clamping during the test and thus are excluded in the analysis. All specimens are subjected to uniaxial tensile test, from which the tensile constitutive model is derived to analyze the parametric influence and deterioration mechanism of corroded steel bars.

**Figure 2:**
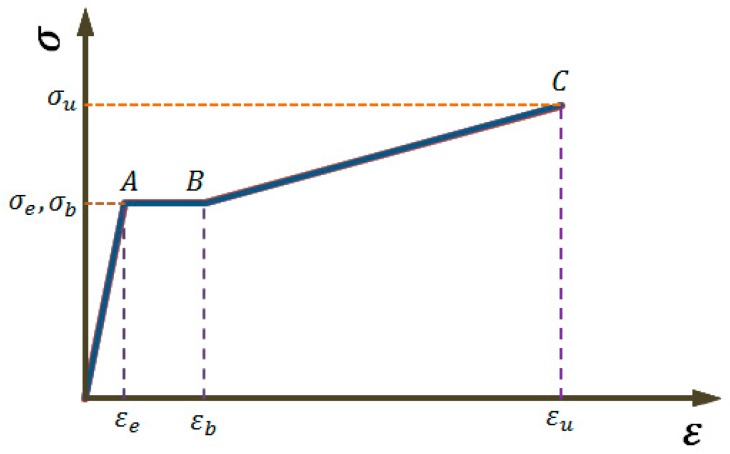
Trilinear simplified constitutive model for mild steel materials.

The tensile constitutive relation of the mild steel usually includes a linear elastic stage, a yielding platform stage, a hardening stage and a descending stage. This paper only examines the first three stages of the steel bar and thus can use a trilinear tensile constitutive model as shown in Figure 2. Among them, the characteristic points *A* ($\varepsilon_{e}$, $\sigma_{e}$), *B* ($\varepsilon_{b}$, $\sigma_{b}$), and *C* ($\varepsilon_{u}$, $\sigma_{u}$) are the tensile yield point, the end point of the yielding platform, and the end point of the hardening stage, respectively. These characteristic points can be determined by the constitutive relation of an uncorroded steel bar. $\varepsilon_{e}$, $\varepsilon_{b}$, and $\varepsilon_{u}$ are the yield strain, hardening strain, and ultimate strain, $\sigma_{e}$, $\sigma_{b}$, and $\sigma_{u}$ are their corresponding stresses, respectively. $\sigma_{e}=\sigma_{b}$. Thus,

Elastic Modulus:

(1) $$E=\frac{\sigma_{e}}{\varepsilon_{e}}$$

The slope of hardening stage:

(2) $$k=\frac{\sigma_{u}-\sigma_{b}}{\varepsilon_{u}-\varepsilon_{b}}$$

The length of yield platform:

(3) $$\Delta \varepsilon_{b}=\varepsilon_{b}-\varepsilon_{e}$$

**Figure 3:**
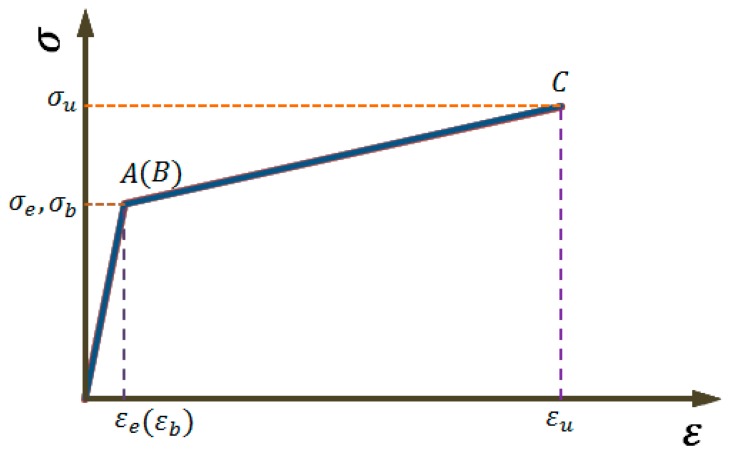
Bilinear simplified constitutive model for mild steel materials [21].

Compared with the bilinear model proposed in [21] (see Figure 3), the trilinear model used in the present study has included a yielding platform, which more accurately reflects the constitutive characteristics of the steel material. 

### 2.2. Simplified Model of the Corroded Steel

**Figure 4:**
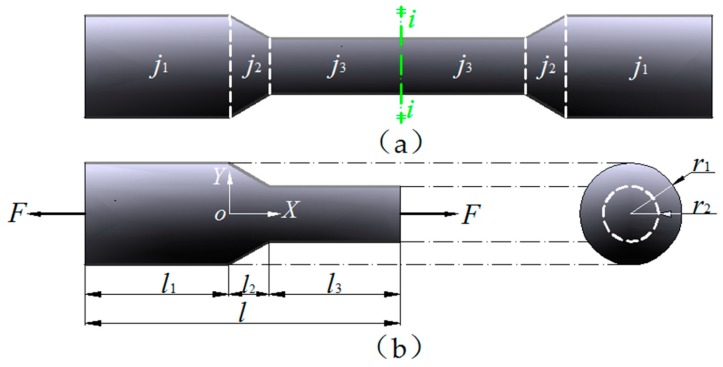
The physical model for corroded steel bars. (**a**) The geographical model of intact steel bars (**b**) coordinates and natations of the half model.

Based on the assumption that the remaining material of the steel bar are unaffected by steel corrosion [13,21,22], the shape of the corroded bar is formed by mechanical slotting, therefore the residual cross-sectional shape of the steel bar after corrosion is simulated. Mechanical slotting is carried out in the uncorroded steel bar shown in Figure 1, and detailed information of slotting is given in Figure 4. In Figure 4a, the corroded steel bar is composed of three parts: a uncorroded part $\left(j_{1}\right)$, a corroded part $\left(j_{2}\right)$ with variable cross section and a uniform corroded part $\left(j_{3}\right)$; the whole bar has a circular section and is axisymmetric, wherein the radii of part $j_{1}$ and $j_{3}$ are $r_{1}$ and $r_{3}$, respectively, and the radius of part $j_{2}$ is linearly reduced from $r_{1}$ to $r_{3}$. The corroded steel bar is assumed to be symmetrical about the vertical axis i−i. Therefore, the study of the constitutive relationship of the corroded steel bar under axial tensile force *F* can be based on the half physical model as shown in Figure 4b, where $l_{1},l_{2},l_{3}$ are the axial original lengths of the three parts ($j_{1}$,$\;j_{2}$,$\;j_{3}$), respectively. The total length (l) of the half physical model is calculated by summing up the lengths of the three parts:

(4) $$l=l_{1}+l_{2}+l_{3}$$

To characterize the radius and area of the part $j_{2}$, a rectangular coordinate system (XoY) is established. The origin o is the center of the interface between the part $j_{1}$ and part $j_{2}$, and the radius $r_{2}\left(x\right)$ can be expressed as $r_{2}\left(x\right)=r_{1}-\left(\left(r_{1}-r_{3}\right)/l_{2}\right)x$. Therefore, the cross-sectional areas of three parts ($j_{1}$, $j_{2}$, $j_{3}$) of the corroded steel bar are

(5) $$A_{1}=\pi r_{1}{}^{2},A_{2}\left(x\right)=\pi {\left(r_{1}-\frac{r_{1}-r_{3}}{l_{2}}x\right)}^{2},\;A_{3}=\pi r_{3}{}^{2}$$

They satisfy the following formulas.

(6) $$A_{1}=\pi r_{1}{}^{2}\geq A_{2}\left(x\right)\geq A_{3}=\pi r_{3}{}^{2}$$

In Figure 4b, the elongation of part $j_{1}$, $j_{2}$,$\;j_{3}$ under the action of tensile force (*F*) is $\Delta l_{1}$,$\;\Delta l_{2}$,$\;\Delta l_{3}$ respectively, and so the total elongation (Δl) of the corroded steel bar in the original length (l) is the sum of elongations of each part [21],

(7) $$\Delta l=\Delta l_{1}+\Delta l_{2}+\Delta l_{3}$$

Thus, the total strain of the corroded steel bar ($\bar{\varepsilon }$) can be defined as

(8) $$\bar{\varepsilon }=\frac{\Delta l}{l}$$

The nominal stress of the corroded steel bar (σ) can be defined as

(9) $$\sigma =\frac{F}{A}$$

where $A=A_{1}=\pi r_{1}{}^{2}$, A is the original cross-sectional area of the uncorroded steel bar.

## 3. Analytical Model of Relationship of the Corroded Steel Bar

If $A_{1}=A_{3}$, it means that the steel bar is not corroded. For corroded steel bars, $A_{1}>A_{3}$, we can see from Equations (10)–(13) that among $F_{e1}$,$\;F_{e3}$,$\;F_{u1}$,$F_{u3}$, $F_{e3}$ is the smallest and $F_{u1}$ is the largest, and the value of $F_{e1}$ and $F_{u3}$ depends on the degree of corrosion of the steel bar.

(10) $$F_{e1}=\sigma_{e}A_{1}$$

(11) $$F_{u1}=\sigma_{u}A_{1}$$

(12) $$F_{e3}=\sigma_{e}A_{3}$$

(13) $$F_{u3}=\sigma_{u}A_{3}$$

where $F_{e1}$ and $F_{u1}$ are the yield and ultimate loads of the uncorroded part $\left(j_{1}\right)$, respectively; $F_{e3}$ and $F_{u3}$ are the yield and ultimate loads of the uniform corroded part $\left(j_{3}\right)$, respectively.

Therefore, the analytical formulas of the constitutive relation of the corroded steel bar can be respectively introduced in the form of three cases.

Case I: $F_{e1}<F_{u3}$, light corrosion

(14) $$\bar{\varepsilon }=\frac{\Delta l}{l}=\frac{F}{El}\left(\frac{l_{1}}{A_{1}}+\frac{l_{3}}{A_{3}}\right)+\int_{0}^{l_{2}}\frac{F}{ElA_{2}\left(x\right)}dx\;\mathrm{When}\;F_{e3}>F\geq 0$$

(15) $$\bar{\varepsilon }=\frac{\Delta l}{l}=\varepsilon_{e}\frac{A_{3}}{A_{1}}\cdot \frac{l_{1}}{l}+\left[\varepsilon_{e},\varepsilon_{b}\right]\frac{l_{3}}{l}+\int_{0}^{l_{2}}\frac{\varepsilon_{e}A_{3}}{lA_{2}\left(x\right)}dx,\;\mathrm{When}\;F=F_{e3}$$

$$\bar{\varepsilon }=\frac{\Delta l}{l}=\frac{F}{EA_{1}}\cdot \frac{l_{1}}{l}+\left(\varepsilon_{b}+\frac{F-\sigma_{e}A_{3}}{A_{3}k}\right)\cdot \frac{l_{3}}{l}+\frac{1}{l}\int_{0}^{x_{0}}\frac{F}{EA_{2}\left(x\right)}dx\;+\frac{1}{l}\int_{x_{0}}^{l_{2}}\left(\varepsilon_{b}+\frac{F-\sigma_{e}A_{2}\left(x\right)}{A_{2}\left(x\right)k}\right)dx,$$

(16) $$\mathrm{When}\;F_{e1}>F>F_{e3}$$

$$\bar{\varepsilon }=\frac{\Delta l}{l}=\left[\varepsilon_{e},\varepsilon_{b}\right]\cdot \frac{l_{1}}{l}+\left(\varepsilon_{b}+\frac{\sigma_{e}\left(A_{1}-A_{3}\right)}{A_{3}k}\right)\cdot \frac{l_{3}}{l}+\frac{1}{l}\int_{0}^{l_{2}}\left(\varepsilon_{b}+\frac{\sigma_{e}\left(A_{1}-A_{2}\left(x\right)\right)}{A_{2}\left(x\right)k}\right)dx$$

(17) $$\mathrm{When}\;F=F_{e1}$$

$$\bar{\varepsilon }=\frac{\Delta l}{l}=\varepsilon_{b}+\frac{F-\sigma_{e}A_{1}}{A_{1}k}\cdot \frac{l_{1}}{l}+\frac{F-\sigma_{e}A_{3}}{A_{3}k}\cdot \frac{l_{3}}{l}+\int_{0}^{l_{2}}\frac{F-\sigma_{e}A_{2}\left(x\right)}{A_{2}\left(x\right)kl}dx$$

(18) $$\mathrm{When}\;F_{u3}>F>F_{e1}$$

$$\bar{\varepsilon }=\frac{\Delta l}{l}=\varepsilon_{b}+\frac{\sigma_{u}A_{3}-\sigma_{e}A_{1}}{A_{1}k}\cdot \frac{l_{1}}{l}+\left(\varepsilon_{u}-\varepsilon_{b}\right)\cdot \frac{l_{3}}{l}+\int_{0}^{l_{2}}\frac{\sigma_{u}A_{3}-\sigma_{e}A_{2}\left(x\right)}{A_{2}\left(x\right)kl}dx$$

(19) $$\mathrm{When}\;F=F_{u3}$$

where [$\varepsilon_{e},\varepsilon_{b}$] represents the strain interval of the yielding platform of the mild steel shown in Figure 2, and its length is given by Equation (3).

Case II: $F_{e1}=F_{u3}$, critical corrosion.

$$\bar{\varepsilon }=\frac{\Delta l}{l}=\frac{F}{El}\left(\frac{l_{1}}{A_{1}}+\frac{l_{3}}{A_{3}}\right)+\int_{0}^{l_{2}}\frac{F}{ElA_{2}\left(x\right)}dx$$

(20) $$\mathrm{When}\;F_{e3}>F\geq 0$$

$$\bar{\varepsilon }=\frac{\Delta l}{l}=\varepsilon_{e}\frac{A_{3}}{A_{1}}\cdot \frac{l_{1}}{l}+\left[\varepsilon_{e},\varepsilon_{b}\right]\frac{l_{3}}{l}+\int_{0}^{l_{2}}\frac{\varepsilon_{e}A_{3}}{lA_{2}\left(x\right)}dx$$

(21) $$\mathrm{When}\;F=F_{e3}$$

$$\bar{\varepsilon }=\frac{\Delta l}{l}=\frac{F}{EA_{1}}\cdot \frac{l_{1}}{l}+\left(\varepsilon_{b}+\frac{F-\sigma_{e}A_{3}}{A_{3}k}\right)\cdot \frac{l_{3}}{l}+\frac{1}{l}\int_{0}^{x_{0}}\frac{F}{EA_{2}\left(x\right)}dx\;+\frac{1}{l}\int_{x_{0}}^{l_{2}}\left(\varepsilon_{b}+\frac{F-\sigma_{e}A_{2}\left(x\right)}{A_{2}\left(x\right)k}\right)dx$$

(22) $$\mathrm{When}\;F_{u3}>F>F_{e3}$$

$$\bar{\varepsilon }=\frac{\Delta l}{l}=\left[\varepsilon_{e},\varepsilon_{b}\right]\cdot \frac{l_{1}}{l}+\varepsilon_{u}\cdot \frac{l_{3}}{l}+\frac{1}{l}\int_{0}^{l_{2}}\left(\varepsilon_{b}+\frac{\sigma_{e}\left(A_{1}-A_{2}\left(x\right)\right)}{A_{2}\left(x\right)k}\right)dx$$

(23) $$\mathrm{When}\;F=F_{u3}=F_{e1}$$

Case III: $F_{e1}>F_{u3}$, severe corrosion.

$$\bar{\varepsilon }=\frac{\Delta l}{l}=\frac{F}{El}\left(\frac{l_{1}}{A_{1}}+\frac{l_{3}}{A_{3}}\right)+\int_{0}^{l_{2}}\frac{F}{ElA_{2}\left(x\right)}dx$$

(24) $$\mathrm{When}\;F_{e3}>F\geq 0$$

$$\bar{\varepsilon }=\frac{\Delta l}{l}=\varepsilon_{e}\frac{A_{3}}{A_{1}}\cdot \frac{l_{1}}{l}+\left[\varepsilon_{e},\varepsilon_{b}\right]\frac{l_{3}}{l}+\int_{0}^{l_{2}}\frac{\varepsilon_{e}A_{3}}{lA_{2}\left(x\right)}dx$$

(25) $$\mathrm{When}\;F=F_{e3}$$

$$\bar{\varepsilon }=\frac{\Delta l}{l}=\frac{F}{EA_{1}}\cdot \frac{l_{1}}{l}+\left(\varepsilon_{b}+\frac{F-\sigma_{e}A_{3}}{A_{3}k}\right)\cdot \frac{l_{3}}{l}+\frac{1}{l}\int_{0}^{x_{0}}\frac{F}{EA_{2}\left(x\right)}dx\;+\frac{1}{l}\int_{x_{0}}^{l_{2}}\left(\varepsilon_{b}+\frac{F-\sigma_{e}A_{2}\left(x\right)}{A_{2}\left(x\right)k}\right)dx$$

(26) $$\mathrm{When}\;F_{u3}>F>F_{e3}$$

$$\bar{\varepsilon }=\frac{\Delta l}{l}=\varepsilon_{u}\frac{A_{3}}{A_{1}}\cdot \frac{l_{1}}{l}+\varepsilon_{u}\cdot \frac{l_{3}}{l}+\frac{1}{l}\int_{0}^{x_{0}}\varepsilon_{u}\frac{A_{3}}{A_{2}\left(x\right)}dx+\frac{1}{l}\int_{x_{0}}^{l_{2}}\left(\varepsilon_{b}+\frac{\sigma_{u}A_{3}-\sigma_{e}A_{2}\left(x\right)}{A_{2}\left(x\right)k}\right)dx$$

(27) $$\mathrm{When}\;F=F_{u3}$$

where $x_{0}=\left(r_{1}-\sqrt{F/\sigma_{e}\pi }\right)l_{2}/\left(r_{1}-r_{3}\right)$, $x=x_{0}$ indicates the elasticity and yield interface of the part $j_{2}$ of the corroded steel bar.

The strength of the corroded steel bar is calculated by Equation (9), and the original cross-sectional area of the uncorroded steel bar is used for convenience, and the simplified stress–strain constitutive curves of steel bars under three cases can be given by combining Equations (14–19), (20–23), or (24–27).

## 4. Tensile Experiments of Corroded Steel Bars

### 4.1. Specimens

The experimental specimens used in this paper include 11 corroded steel bars and 1 uncorroded steel bar. The total length of the specimen is 260 mm, which includes the length of the two end portions (2 × 70 mm) and gives an effective length of 120 mm with original radius $r_{1}=$ 18 mm. By mechanically grooving the effective part of the uncorroded specimen, specimens with different corroded conditions are obtained (see Figure 4a). Since all the specimens are vertically symmetrical, corresponding to Figure 4b, the geometric parameters of the left half of the symmetry axis of effective parts of the 12 corroded specimens with different corroded conditions are listed in Table 1. Among them, the specimen AS1-1 is the uncorroded steel bar and the other 11 specimens have different degrees of corrosion, represented by different corroded lengths and slope angles. The slope angle *β* satisfies

(28) $$\tan\beta =\Delta r/l_{2}$$

where

(29) $$\Delta r=r_{1}-r_{3}$$

### 4.2. Experiment 

The uniaxial tensile tests are conducted according to the “Metal Material Tensile Test: Room Temperature Test Method” procedure. The tests are carried out at a speed of 3 mm/min using a 300 kN MTS universal test machine [21]. The tensile force (*F*) is recorded by force sensor attached to the grips and the elongation length (ΔL) of the specimen is obtained by an electronic YBC type extensometer, and its range is matched with the length of the effective part.

## 5. Results and Discussion 

### 5.1. The Tensile Constitutive Curve of the Steel Bars 

The measured tensile force (*F*) and elongation (ΔL) can be converted into constitutive stress and strain relation according to Equations (8) and (9). Note that in the experiments it measures the elongation of the whole effective length of the specimen. Thus, Equation (8) can be rewritten as $\bar{\varepsilon }=\frac{2\Delta l}{2l}=\frac{\Delta L}{L}$, where *L* = 120 mm is the effective length of the speciemn.

**Figure 5:**
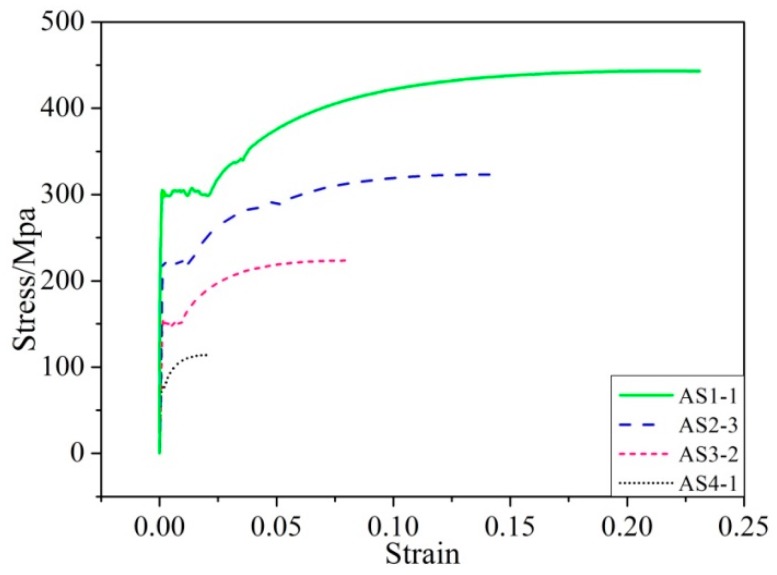
Experimentally obtained constitutive relationship of partial corroded steel bars.

Figure 5 shows the stress–strain curves obtained by uniaxial tensile tests of three partially corroded steel bars. For the purpose of comparison, the stress–strain curve of an uncorroded steel bar is also superimposed in Figure 5. It can be clearly seen from Figure 5 that both the strength and ductility of the steel bar decrease with the increase of the degree of corrosion.

The coordinates of the key points *A*, *B* and *C* of the simplified trilinear constitutive model of the mild steel shown in Figure 2 can be obtained from the stress–strain curve of the uncorroded steel bar in Figure 5:

(30) $$\varepsilon_{e}=0.0009\;\;\;\;\;\sigma_{e}=311\mathrm{Mpa}$$

(31) $$\varepsilon_{b}=0.0292\;\;\;\;\;\sigma_{b}=311\mathrm{Mpa}$$

(32) $$\varepsilon_{u}=0.2325\;\;\;\;\;\sigma_{u}=433\mathrm{Mpa}$$

Theoretical solutions could be obtained by substituting Equations (30)–(32) into the analytical formulas of the average strain of corroded steel bars for various different cases.

### 5.2. Comparison of the Theoretical Solution and Experimental Result

To facilitate the comparison between the theoretical solutions and experimental results, the concept of relative error is introduced here. The calculation formula is listed below.

(33) $$\mathrm{Relative}\;\mathrm{error}\;=\;\frac{\left|\mathrm{Theoretical}\;\mathrm{solution}-\mathrm{Experimental}\;\mathrm{result}\right|}{\mathrm{Experimental}\;\mathrm{result}}$$

**Figure 6:**
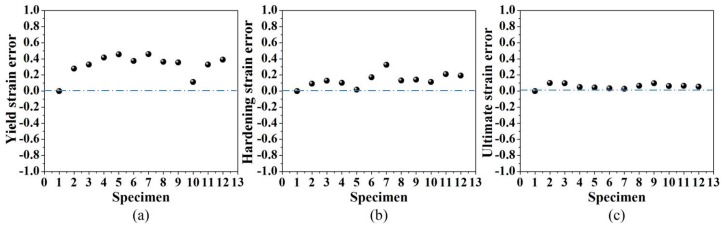
The relative error of theoretical strains (**a**) Yield strain error (**b**) Hardening strain error (**c**) Ultimate strain error.

The relative errors of yield strain, hardening strain and ultimate strain are shown in Figure 6. It could be seen from Figure 6 that the relative errors for ultimate strain and hardening strain are small, indicating good agreement between the theoretical results and experimental data. However, the relative errors for yield strain are large. This is because the measured yield strains are extremely small, which leads a large fluctuation (see Figure 6a). However, as the strain value increases, the corresponding error decreases (all below 0.097), as is demonstrated in Figure 6c. 

**Figure 7:**
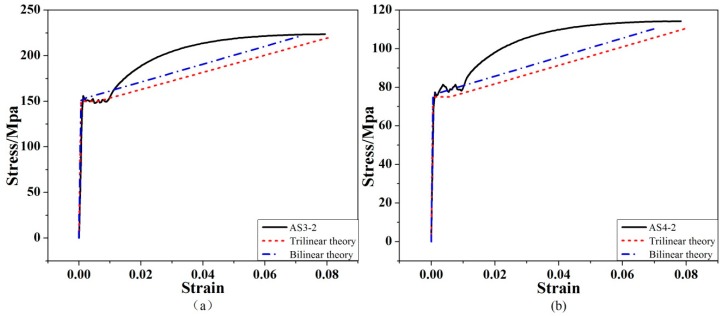
Comparisons of trilinear and bilinear constitutive models with experimentally obtained stress–strain curve (**a**) AS3-2 (**b**) AS4-2.

To further examine the prediction accuracy of the trilinear and bilinear constitutive models, the prediction result from the trilinear constitutive model is calculated by substituting the test values of Equations (30–32) into Equations (14–19), (20–23), or (24–27), and the prediction result of the bilinear model is obtained by substituting Equations (14–19), (20–23), or (24–27) in the case of $\varepsilon_{b}=\varepsilon_{e}$ in Equations (30–32). Figure 7 shows the comparisons of these two models with the experimental stress–strain constitutive curves obtained from two specimens with different cross-sectional corrosion conditions (see Table 1). It is evident from the comparisons that the trilinear model is better than the bilinear model for predicting the yield platform, hardening strain, and ultimate strain of the corroded steel bars. 

### 5.3. The Attenuation Law of Ultimate Strength 

To characterize the damage degree of corroded steel bars, the loss rate $\left(\xi_{A}\right)$ of cross-sectional area is introduced:

(34) $$\xi_{A}=1-A_{3}/A_{1}$$

When $F_{e1}=F_{u3}$, $\sigma_{e}A_{1}=\sigma_{u}A_{3}$ can be obtained according to Equations (10) and (13). This state can be defined as “critical corrosion”, and the corresponding loss rate of cross-sectional area is defined as critical loss rate $\left[\xi_{A}\right]$:

(35) $$\left[\xi_{A}\right]=1-\sigma_{e}/\sigma_{u}$$

When $F_{e1}<F_{u3}$, $\sigma_{e}A_{1}<\sigma_{u}A_{3}$ can be obtained according to Equations (10) and (13). Thus, $\xi_{A}<\left[\xi_{A}\right]$ can be derived and this state can be defined as “light corrosion”. 

When $F_{e1}>F_{u3}$, $\sigma_{e}A_{1}>\sigma_{u}A_{3}$ can be obtained according to Equations (10) and (13). The loss rate in this state is $\xi_{A}>\left[\xi_{A}\right]$, and this state can be defined as “severe corrosion”. 

The above three states correspond to the case one, two and three, respectively. As seen in Figure 5, the loss rate of cross-sectional area increases, and the strength of the corroded steel bar is gradually reduced. The ultimate load of the corroded steel bar can be calculated by

(36) $$F_{max}=F_{u3}=\sigma_{u}A_{3}=\left(1-\xi_{A}\right)\sigma_{u}A_{1}$$

The ultimate strength of the corroded steel bar can be obtained by

(37) $$\bar{\sigma_{u}}=F_{max}/A_{1}=\left(1-\xi_{A}\right)\sigma_{u}$$

**Figure 8:**
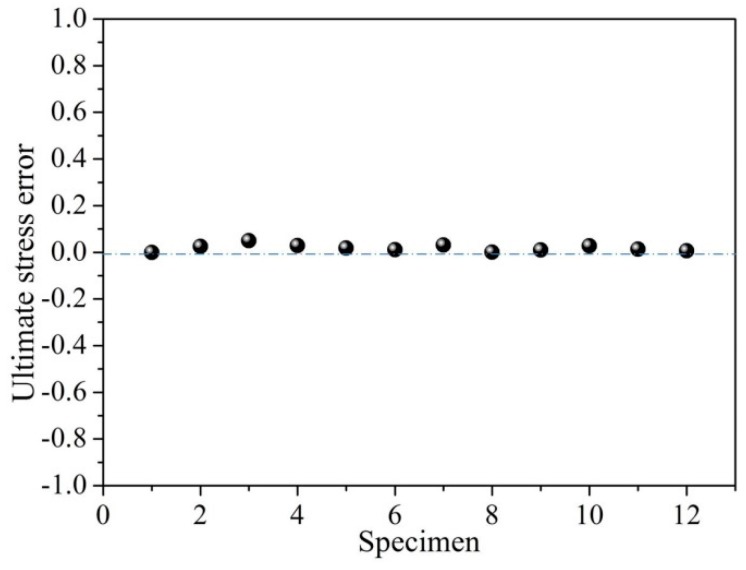
Relative error of theoretical ultimate stress.

It can be concluded that the ultimate strength of the corroded steel bar decreases with the increase of the loss rate of cross-sectional area of the corroded bar. According to Equation (33), the relative error of the predicted ultimate strength can be obtained (see Figure 8). It can be observed from Figure 8 that the predicted value is very close to the experimental result, which demonstrates the appropriateness of the present model.

### 5.4. The Attenuation Law of Ductility 

Figure 5 shows not only the decrease in strength but also the reduction in the ductility, the latter of which is mainly reflected in the shortening of the yield platform and the reduction of the ultimate strain. When a corroded steel bar is in the state of light corrosion (case I), it can be seen from Equations (15) and (17) that the length of the yield platform is $\left[\varepsilon_{e},\varepsilon_{b}\right]\frac{l_{3}}{l}+\left[\varepsilon_{e},\varepsilon_{b}\right]\frac{l_{1}}{l}$, which is why the steel bar still has a relatively long length of yielding platform. However, when a steel bar is severely corroded (case III), the length of the yielding platform of the corroded bar is obtained by Equation (25) as $\left[\varepsilon_{e},\varepsilon_{b}\right]\frac{l_{3}}{l}$, at which time the length of the yielding platform of the corroded steel bar is only relevant to the length of the uniform corroded part $l_{3}$. When the steel corrosion is pitting, that is, $l_{3}\to 0$, the yielding platform of the corroded bar will disappear. 

**Figure 9:**
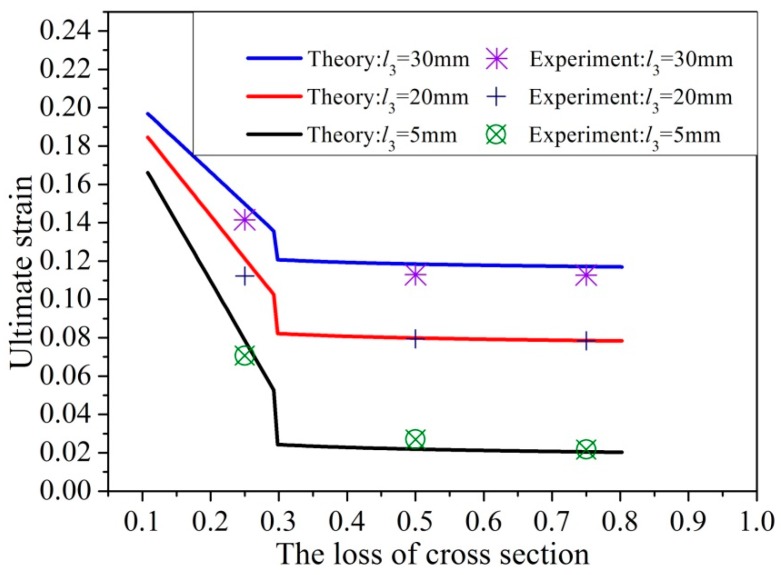
Comparison of experimental tensile results and theoretical predictions.

According to Equations (19) and (27), the ultimate strain of the corroded steel bar can be calculated so that the relationship of the ultimate strain and the loss rate of cross-sectional area of the corroded steel bar can be described. The results predicted from the present analytical model are shown in Figure 9 for three corroded steel bars with different lengths of $l_{3}$. 

The critical loss rate of corroded steel bars can be calculated by Equation (35) with the value $\left[\xi_{A}\right]=0.3$. When $\xi_{A}<0.3$, the ultimate strain of corroded steel bars decreases linearly with the increase of the loss rate of cross-sectional area; when $\xi_{A}>0.3$, the ultimate strain is almost unchanged. It can be seen from Figure 9 that the theoretical predictions fit well with the experimental data. It is shown that, the longer the length of the uniform corroded part $j_{3}$, the greater the ultimate strain.

### 5.5. Influence of Geometric Parameters of Corroded Steel Bars

#### 5.5.1. Effect of Length $l_{3}$ of the Uniform Corroded Part on Ultimate strain

**Figure 10:**
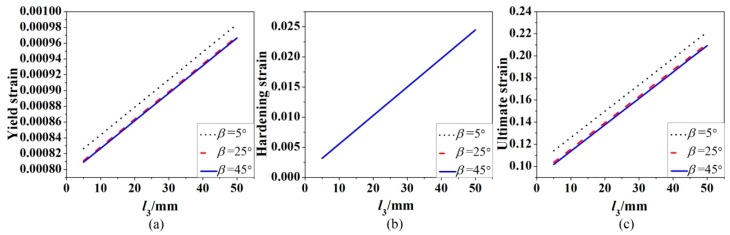
Effect of the length $l_{3}$ of uniform corroded part on yield strain (**a**), hardening strain (**b**), and ultimate strain (**c**) *β* is the slope angle of the corroded part with variable cross-section.

It can be seen from Equations (9–13) that the strength of the corroded steel bar is determined by the strength of the steel material itself and the remaining cross-sectional area of the corroded steel bar, so the length of uniform corroded part does not affect the strength of the corroded steel bar. Equations (14)–(27), under the condition of Δr=1mm, can be used to examine how the length $l_{3}$ of uniform corroded part affects the yield strain, hardening strain and ultimate strain. The corresponding results are shown in Figure 10. It is clear from Figure 10 that all of the three strains increases linearly with the increased length $l_{3}$.

#### 5.5.2. Effect of the Reduction ∆r of the Section Radius

The larger reduction ∆*r* of the cross-section radius is, the larger the loss rate of the cross-sectional area is, and so the strength of the corresponding steel bar will also decrease. The strength variation has been discussed in Section 5.3, and thus is not discussed again here.

**Figure 11:**
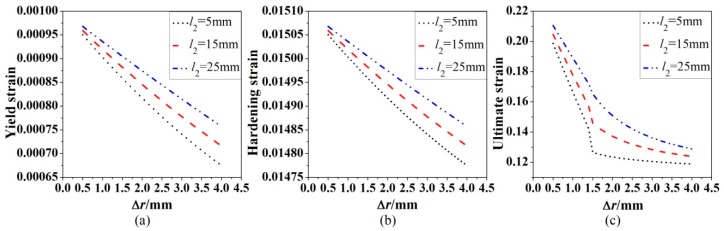
Effect of reduction ∆*r* of the cross-section radius on yield strain (**a**), hardening strain (**b**) and ultimate strain (**c**).

In Equations (14-27), the influence of reduction ∆*r* of the cross-sectional radius on the yield strain, hardening strain and ultimate strain can be theoretically examined. Figure 11 shows the relationships between the three strains of corroded steel bars with three different $l_{2}$ and reduction ∆*r* of the cross-section radius with a fixed uniform corroded length $l_{3}=30\mathrm{mm}$. The yield strain and hardening strain shown in Figure 11a,b decrease with the increase of reduction ∆*r* of the cross-section radius, and the reduction ∆r of the cross-sectional radius pose more impact on the corroded steel bars with smaller $l_{2}$. An interesting phenomenon appears in Figure 11c that as the reduction ∆*r* of the cross-sectional radius increases, the ultimate strength of the corroded steel bar decreases rapidly and then is unaffected by the corrosion reduction ∆*r* of the cross-sectional radius.

#### 5.5.3. The Effect of Slope Angle β of the Corroded Part $j_{2}$

**Figure 12:**
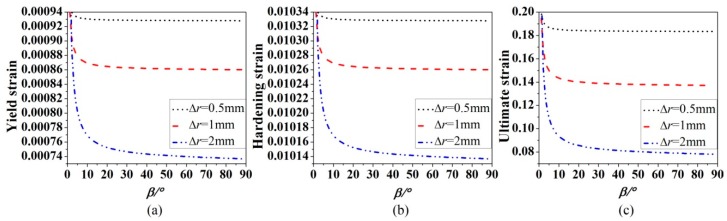
Effect of the slope angle *β* of the corroded part $j_{2}$ on yield strain (**a**), hardening strain (**b**) and ultimate strain (**c**).

The influence of the slope angle *β* on the yield strain, hardening strain and ultimate strain can be theoretically examined using Equations (14–27). Figure 12 shows the relationships between the three strains of corroded bars with different ∆*r* and the slope angle *β* when $l_{3}=20\mathrm{mm}$. It can be seen from Figure 12 that the slope angle *β* has the similar influence on the yield strain, hardening strain and ultimate strain; the three strains in the early stage and decrease sharply with the increase of the slope angle *β*, but almost remain stable in the later stage; the strains of the severe corroded steel bar are more affected by the slope angle *β*.

### 5.6. Discussion

From the above analysis, it can be seen that the strength of the corroded steel bar decreases with the increase of the loss rate of the cross-sectional area, regardless of the corrosion length of the steel bar. When the corroded steel bar is in the state of light corrosion, the steel bar still has a relatively long range of yielding platform. However, when the steel bar is severely corroded, the range of the yielding platform of the corroded bar will be short; when the steel corrosion is pitting corrosion, i.e., $l_{3}\to 0$, the yielding platform of the corroded bar will disappear. The yield strain, hardening strain and ultimate strain of the corroded steel bar increase with the increase of length of the uniform corrosion of the steel bar; the yield strain and hardening strain decrease with the increase of the reduction of the section radius of the steel bar, but as reduction of the section radius increases, the ultimate strain first shows a downward trend and then does not change.

Although this proposed model can characterize mechanical properties of artificial slotted bars with satisfactory accuracy, there are still factors that should be taken into consideration for naturally corroded steel bars. Indeed, the corrosion affects mainly the outer layer of the steel bars which has higher strength than the ferrite core [23]. This spatial heterogeneity was not taken into account in the proposed model and need to be further analyzed in future. Also, contrary to the artificially corroded steel bars, pitting corrosion reduces the cross-sectional area of the steel bars in a localized manner. The necking phenomenon that occurs on the pit holes during the tensile test of the naturally corroded steel bars could decrease the experimented lengthening in steel, which might affect the accuracy of the proposed model. For better prediction of the tensile behavior of the corroded reinforcing bars, these factors should be analyzed carefully in the further research.

## 6. Conclusions

This paper proposes a simplified trilinear model for corroded steel bars, which is well refined so it can quantify more accurately the influence of corrosion on the mechanical properties of corroded steel bars. Based on the proposed trilinear model, the characteristics of a corroded steel bar are examined. The theoretical calculations are validated by the test results of 12 slotted steel bars and the following conclusions can be made.

1. The proposed model can predict the tensile behavior of a corroded steel bar. The prediction for ultimate stress and ultimate strain agree well with the experimental results.
2. Compared to bilinear model, the trilinear model can predict the tensile behavior of a corroded steel bar with higher accuracy especially for light corrosions.
3. The strength of the corroded steel bar decreases with the increase of the loss rate of the cross-sectional area, regardless of the corrosion length of the steel bar.
4. The yield strain, hardening strain and ultimate strain of the corroded steel bar increase with the increase of length of the uniform corrosion of the steel bar.

## Figures and Tables

**Table 1:** Geometric characteristic parameters of specimens of simulated corrosion.

Specimen	Label	$l_{1}\left(\mathrm{mm}\right)$	$l_{2}\left(\mathrm{mm}\right)$	$l_{3}\left(\mathrm{mm}\right)$	$r_{1}\left(\mathrm{mm}\right)$	$r_{3}\left(\mathrm{mm}\right)$	Δr(mm)	*β*
1	AS1-1	60	0	0	9	9	0	0
2	AS2-1	52.9	2.10	5	9	7.79	1.21	30°
3	AS2-2	37.9	2.10	20	9	7.79	1.21	30°
4	AS2-3	27.9	2.10	30	9	7.79	1.21	30°
5	AS3-1	50.43	4.57	5	9	6.36	2.64	30°
6	AS3-2	35.43	4.57	20	9	6.36	2.64	30°
7	AS3-3	25.43	4.57	30	9	6.36	2.64	30°
8	AS3-4	53.48	1.52	5	9	6.36	2.64	60°
9	AS3-5	55	0	5	9	6.36	2.64	90°
10	AS4-1	47.21	7.79	5	9	4.50	4.50	30°
11	AS4-2	32.21	7.79	20	9	4.50	4.50	30°
12	AS4-3	22.21	7.79	30	9	4.50	4.50	30°

## Notation

$\varepsilon_{e}$	Yield strain;
$\varepsilon_{b}$	Hardening strain;
$\varepsilon_{u}$	Ultimate strain;
$\sigma_{e}$	Yield stress;
$\sigma_{b}$	Hardening stress;
$\sigma_{u}$	Ultimate stress;
E	Elastic Modulus;
k	Slope of hardening stage;
$\Delta \varepsilon_{b}$	Length of yield platform;
$j_{1}$	Uncorroded part;
$j_{2}$	Corroded part with variable cross-section;
$j_{3}$	Uniform corroded part;
$r_{1}$	Cross-sectional radius of uncorroded part;
$r_{2}\left(x\right)$	Cross-sectional radius of corroded part with variable cross-section;
$r_{3}$	Cross-sectional radius of uniform corroded part;
$l_{1}$	Axial original length of uncorroded part;
$l_{2}$	Axial original length of corroded part with variable cross-section;
$l_{3}$	Axial original length of uniform corroded part;
l	Total length of the half physical model;
$\Delta l_{1}$	Elongation of uncorroded part;
$\Delta l_{2}$	Elongation of corroded part with variable cross-section;
$\Delta l_{3}$	Elongation of uniform corroded part;
Δl	Total elongation of three parts;
*F*	Axial tensile force;
$\bar{\varepsilon }$	Average strain of the corroded steel bar;
σ	Nominal stress of the corroded steel bar;
A	Original cross-sectional area of the uncorroded steel bar;
$A_{1}$	Cross-sectional area of uncorroded part;
$A_{2}\left(x\right)$	Cross-sectional area of corroded part with variable cross-section;
$A_{3}$	Cross-sectional area of uniform corroded part;
$F_{e1}$	Yield load of uncorroded part;
$F_{u1}$	Ultimate load of uncorroded part;
$F_{e3}$	Yield load of uniform corroded part;
$F_{u3}$	Ultimate load of uniform corroded part;
*β*	Slope angle of corroded part with variable cross-section;
$\xi_{A}$	Loss rate of the cross-sectional area;
$\left[\xi_{A}\right]$	Critical loss rate of the cross-sectional area;
$F_{max}$	Ultimate load of the corroded steel bar;
$\bar{\sigma_{u}}$	Ultimate strength of the corroded steel bar;
∆*r*	Reduction of the cross-section radius;
[$\varepsilon_{e},\varepsilon_{b}$]	Strain interval of the yielding platform of the mild steel;
